# Grey and white matter differences in Chronic Fatigue Syndrome – A voxel-based morphometry study

**DOI:** 10.1016/j.nicl.2017.09.024

**Published:** 2017-09-28

**Authors:** Andreas Finkelmeyer, Jiabao He, Laura Maclachlan, Stuart Watson, Peter Gallagher, Julia L. Newton, Andrew M. Blamire

**Affiliations:** a Institute of Neuroscience, Newcastle University, Newcastle upon Tyne, England, UK; b Aberdeen Biomedical Imaging Centre, University of Aberdeen, Scotland, UK; c Department of Public Health and Community Medicine, Göteborgs Universitet, Göteborg, Sweden; d Institute of Cellular Medicine, Newcastle University, Newcastle upon Tyne, England, UK; e Newcastle Magnetic Resonance Centre, Newcastle University, Newcastle upon Tyne, England, UK

**Keywords:** Chronic Fatigue Syndrome, Voxel-based morphometry, Insula, Amygdala, Midbrain

## Abstract

**Objective:**

Investigate global and regional grey and white matter volumes in patients with Chronic Fatigue Syndrome (CFS) using magnetic resonance imaging (MRI) and recent voxel-based morphometry (VBM) methods.

**Methods:**

Forty-two patients with CFS and thirty healthy volunteers were scanned on a 3-Tesla MRI scanner. Anatomical MRI scans were segmented, normalized and submitted to a VBM analysis using randomisation methods. Group differences were identified in overall segment volumes and voxel-wise in spatially normalized grey matter (GM) and white matter (WM) segments.

**Results:**

Accounting for total intracranial volume, patients had larger GM volume and lower WM volume. The voxel-wise analysis showed increased GM volume in several structures including the amygdala and insula in the patient group. Reductions in WM volume in the patient group were seen primarily in the midbrain, pons and right temporal lobe.

**Conclusion:**

Elevated GM volume in CFS is seen in areas related to processing of interoceptive signals and stress. Reduced WM volume in the patient group partially supports earlier findings of WM abnormalities in regions of the midbrain and brainstem.

## Highlights

• VBM study of patients with Chronic Fatigue Syndrome without depression.
• Patients show increased grey matter in insular cortex and parts of the limbic system.
• Patients show decrease in white matter in midbrain and temporal lobe.
• Findings suggest potentially altered processing of interoceptive signals.

## 1 Introduction

Chronic Fatigue Syndrome (CFS) is a chronic condition of unclear aetiology that is characterised by a variety of diverse symptoms such as chronic, disabling fatigue, unrefreshing sleep, post-exertional malaise, muscle pains and reduced cognitive performance. Brain imaging studies in CFS suggest alterations in brain structure and function in at least subgroups of CFS patients (Natelson, 2013). Previous studies on volumetric brain differences in CFS are scarce and have produced inconsistent findings. An early voxel-based morphometry (VBM) study reported reduction of grey matter (GM) volume in bilateral prefrontal cortex which correlated with reduced functional status (Okada et al., 2004). In contrast, a more recent VBM study showed regional reductions in GM volume only in occipital and parahippocampal regions, as well as white matter (WM) volume reductions in occipital regions (Puri et al., 2012). An overall reduction in supratentorial WM volume has been reported, with no difference in cortical GM volume (Zeineh et al., 2015). Overall reductions in GM volume have been reported in two independent cohorts of CFS patients (de Lange et al., 2005), but this was not replicated in a subsequent study by the same group (van der Schaaf et al., 2016) or in recent studies by another group (Barnden et al., 2011, Barnden et al., 2016). These studies also found no significant regional GM or WM volume differences between CFS patients and controls. Instead, significant differences were found in the associations of brain MRI measures (regional volume, scaled image intensity) and various cardiovascular parameters (heart rate, blood pressures) between CFS patients and controls, which were largely confined to regions of predominant WM, and with several consistent and strong findings in the brainstem (Barnden et al., 2011, Barnden et al., 2016).

Various factors may have contributed to these disparate findings in the literature. They include the size and make-up of the samples as well as differences in imaging and analysis methodology. Apart from the most recent study (van der Schaaf et al., 2016), patient sample sizes of all previous studies are small to moderate with < 30 patients in all studies. Furthermore, not all studies explicitly excluded patients with a psychiatric comorbidity, which may have affected findings. Differences in analysis methodology included the identity and version of the processing software, different choices of the size of the smoothing kernel and the selection of covariates for the statistical model. For instance, several previous studies have included segment volume (as opposed to total intracranial volume) as covariate in their models. This may have reduced the power to detect local differences as such local differences would have also influenced the overall segment volume, and thus systematic differences in local volume could be falsely attributed to the segment volume covariate (Henley et al., 2010).

Given the heterogeneity of previous findings, the current study investigated GM and WM volume differences between CFS patients and healthy controls with a moderately sized sample of CFS patients which has been meticulously screened for psychiatric comorbidities. The VBM analysis of the present study will rely on a recent implementation of the segmentation and normalization procedures (Gaser and Dahnke, 2016), which incorporates several major changes in the image processing algorithms that are aimed to improve the quality of the resulting normalized tissue segments over previous implementations.

## 2 Methods

### 2.1 Patients

A group of 42 patients with CFS (32 female, mean age 45.2) participated in this study as part of a larger project investigating the role of autonomic dysfunctions in CFS. They were recruited via the Newcastle and North Tyneside National Health Service (NHS) Clinical CFS Service. Consecutive patients attending the clinic were provided with a Patient Information Sheet and invited to contact the research team if they were willing to be involved. Participants were not selected according to any criteria other than fulfilling the Fukuda diagnostic criteria of CFS (Fukuda et al., 1994). During an initial screening visit using the Structured Clinical Interview for the Diagnostic and Statistical Manual for Mental Disorders (version IV; SCID-IV, (First et al., 2002)) patients that screened positive for a current or past major depressive episode were excluded from further participation in the study. None of the participants that were scanned fulfilled diagnostic criteria for any other axis-I disorder.

A group of 30 healthy volunteers were recruited via notices provided in the hospital and University together with a distribution of posters via the local patient support groups. One third were recruited as part of the above mentioned CFS study, with the remaining two thirds being recruited as healthy volunteers in a clinical trial investigating treatment-resistant major depression (McAllister-Williams et al., 2016). Recruitment for both studies happened during approximately the same time period. Both studies had similar requirements in terms of the age and sex distribution of the healthy volunteer groups, and enforced the same set of relevant inclusion and exclusion criteria for healthy volunteers, i.e. absence of any psychiatric or major physical health conditions.

All participants provided written informed consent. The study was conducted in accordance with the Declaration of Helsinki. The study received a favourable ethical opinion from the local NHS Research Ethics Committee.

### 2.2 MR imaging and processing

All participants were scanned on the same 3T Achieva® (Philips Healthcare, Best, NL) MR scanner with an 8 channel head coil for signal detection at the Newcastle Magnetic Resonance Centre. A standard clinical T1-weighted anatomical scan was collected using a 3D MPRAGE sequence (TE = 4.6 ms, TR = 8.3 ms, flip angle = 8°, 3D-acquisition, FOV: 240 mm (AP) × 216 mm (FH) × 180 mm (LR), 1 mm isotropic voxel size). Images were manually reoriented to place their native-space origin at the anterior commissure. Images were then pre-processed using the Computational Anatomy Toolbox (CAT12) (Gaser and Dahnke, 2016) for SPM12 (www.fil.ac.uk/spm/) in Matlab R2014b (The Mathworks, Inc., Natick, MA, USA). This included bias-field and noise removal, skull stripping, segmentation into grey and white matter, and finally normalization to MNI space using DARTEL to a 1.5 mm isotropic adult template provided by the CAT12 toolbox. Some parameters of these steps were slightly adjusted from their default values, as initial segmentations had occasionally resulted in misclassification of the meninges and transverse sinus as grey matter. Intensity modulation of the normalized tissue segments accounted for both global affine transformations and local warping. The toolbox further provided ratings of image data quality, which were used to identify problems with individual images. These assess basic image properties, noise and geometric distortions (e.g. due to motion) and combine them into a weighted image quality rating (IQR). As a result the scan of one control participant was excluded from further analysis as it had an IQR above 2.7, which was > 5 standard deviations above the mean rating of the sample (1.986 ± 0.133). A further check of sample homogeneity indicated large discrepancies between the overall sample and one (additional) control participant, who was subsequently removed, leaving 28 of 30 healthy control participants for final analysis. Grey and white matter segments were then spatially smoothed using a 6 mm FWHM Gaussian smoothing kernel.

### 2.3 Statistical analysis

Overall volumes of GM, WM, CSF and their sum (total intracranial volume; TIV), as estimated by the CAT12 toolbox, were compared using independent sample *t*-tests in SPSS version 21 (IBM Corp., Armonk, NY, USA). Exploratory analyses in the patient group related segment volume and TIV to several illness characteristics using simple and multiple regression analyses: total and subscale scores of the Fatigue Impact Scale (FIS) (Fisk et al., 1994), total score on the Cognitive Failures Questionnaire (CFQ) (Broadbent et al., 1982), total score on the 31-item version of the Composite Autonomic Symptom Scale (COMPASS-31) (Sletten et al., 2012), self-reported age at onset and derived duration of symptoms.

Voxel-based group comparisons of smoothed GM and WM segments were performed using permutation tests using the randomise command (Winkler et al., 2014) of the FMRIB Software Library (Jenkinson et al., 2012) with the threshold-free cluster enhancement (TFCE) option (Smith and Nichols, 2009). This approach has been shown to be an effective method to deal with smoothness nonstationarity in VBM analyses (Salimi-Khorshidi et al., 2011) and its effects on cluster-based inference (Eklund et al., 2016). Tests included age, sex and total intracranial volumes as confound regressors. The number of permutations was set to 10,000. The TFCE method with randomisation testing produces maps of cluster-enhanced *t* statistics (pseudo-*t*) and maps of p-values that correct for multiple comparisons (across space) (Smith and Nichols, 2009). Voxels were considered to be significantly different between groups when their value on these maps was p < 0.05.

## 3 Results

Table 1 shows basic participant characteristics and overall brain segment volume measures. There were no differences between the groups in terms of age (patients: M = 45.6, SD = 11.7; controls: M = 48.4, SD = 11.3, *t*(68) = 0.984, p = 0.329) or gender (patients: 32f/10 m; controls: 19f/9 m, χ^2^ = 0.590, p = 0.442). Controls showed slightly larger total intracranial volume (TIV) than patients (patients: M = 1486.5 ml, SD = 129.6; controls: M = 1559.5 ml, SD = 148.4; *t*(68) = 2.179, p = 0.033). This difference was driven by a difference in overall WM volume (patients: M = 516.5 ml, SD = 57.6; controls: M = 558.7 ml, SD = 57.6; *t*(68) = 3.007, p = 0.004). There was also a trend for larger volume of cerebrospinal fluid (CSF) in controls (patients: M = 303.4 ml, SD = 61.4; controls: M = 332.2 ml, SD = 76.1; *t*(68) = 1.744, p = 0.086). Absolute GM volumes did not differ between the CFS group (M = 666.5 ml, SD = 57.8) and the control group (M = 668.6 ml, SD = 60.5; *t*(68) = 0.140, p = 0.889). Given the group difference in TIV, the analysis of GM, WM and CSF volumes were repeated using Analysis of Covariance (ANCOVA) while covarying for TIV. Patients had higher adjusted GM volume (M_adj_ = 675.7 ml) than controls (M_adj_ = 654.8 ml; F(1,67) = 4.144, p = 0.046), and lower adjusted WM volume (CFS: M_adj_ = 527.2 ml; HC: M_adj_ = 542.7 ml; F(1,67) = 4.717, p = 0.033). There was no significant difference in adjusted CSF volume (p = 0.696).

**Table 1:** Group demographics and overall segment volumes.

	ME/CFS	Control	Test statistic	p-Value
Age (years)	45.6 (11.7)	48.4 (11.3)	*t* = 0.984	0.33
Gender	32f/10m	19f/9m	χ^2^ = 0.590	0.44
TIV (ml)	1486.5 (129.6)	1559.5 (148.4)	*t* = 2.179	**0.033**
GM volume (ml)				
Absolute (M, SD)	666.5 (57.8)	668.6 (60.5)	*t* = 0.140	0.889
TIV-adjusted (M, SE)	675.7 (6.35)	654.8 (7.83)	F = 4.144	**0.046**
WM volume (ml)				
Absolute (M, SD)	516.5 (57.6)	558.7 (57.6)	*t* = 3.007	**0.004**
TIV-adjusted (M, SE)	527.2 (4.44)	542.7 (5.47)	F = 4.717	**0.033**
CSF volume (ml)				
Absolute (M, SD)	303.4 (61.4)	332.2 (76.1)	*t* = 1.744	*0.086*
TIV-adjusted (M, SE)	312.8 (8.08)	318.1 (9.96)	F = 0.165	0.696

p -value in bold for p < 0.05 and in italics for p < 0.1.

**Fig. 1:**
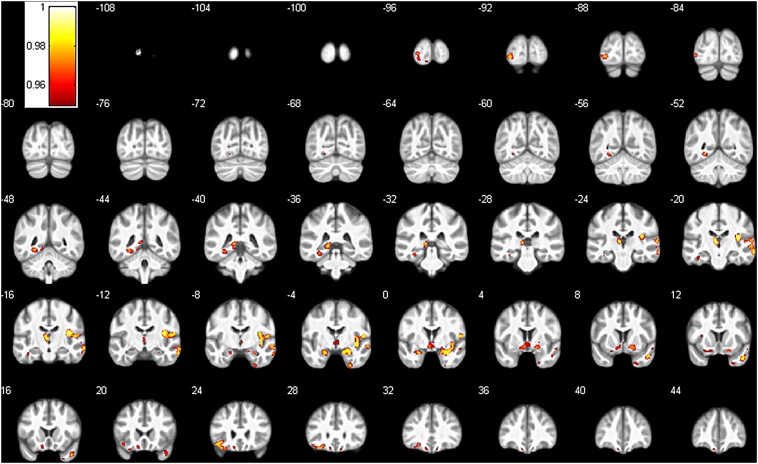
Areas of significantly increased grey matter in the CFS group compared to the control group. Results show the FWE-corrected p-value map (1-p, p < 0.05) overlaid on the average of all normalized images of the entire sample. Numbers indicate y-coordinate of the slice in MNI space.

The VBM analysis showed significant differences in GM volume in several regions throughout the brain (see Fig. 1, Table 2). Patients showed higher GM in widespread areas of the right temporal lobe including the insular cortex, in various subcortical areas such as the bilateral amygdala, putamen, thalamus and hippocampus, parts of the left inferior frontal lobe and left occipital lobe. There were no significant areas with reduced GM volume in the patient group.

**Table 2:** VBM grey matter results.

Cluster index	n Voxel	Pseudo-*t*^a^	x	y	z	Label^b^
1	3368	4.93	37.5	− 16.5	18.0	R central operculum
		4.88	54.0	− 1.5	10.5	R central operculum
		4.65	27.0	− 1.5	− 21.0	R amygdala
		4.60	40.5	− 3.0	− 6.0	R posterior insula
		4.16	55.5	− 12.0	15.0	R central operculum
		4.12	66.0	− 19.5	− 10.5	R middle temporal gyrus
		4.12	21.0	10.5	− 18.0	R posterior orbital gyrus
		4.00	18.0	− 3.0	− 19.5	R amygdala
		3.92	64.5	− 22.5	9.0	R cerebral white matter
		3.88	16.5	7.5	− 10.5	R Putamen
		3.83	63.0	− 10.5	− 22.5	R middle temporal gyrus
		3.80	61.5	− 19.5	− 1.5	R superior temporal gyrus
		3.80	49.5	− 15.0	10.5	R transverse temporal gyrus
		3.77	34.5	0.0	− 16.5	R cerebral white matter
		3.68	58.5	− 7.5	− 7.5	R superior temporal gyrus
		2.90	21.0	6.0	− 3.0	R putamen
2	868	5.69	− 4.5	− 19.5	7.5	L thalamus proper
		4.18	− 13.5	− 37.5	1.5	L hippocampus
		4.15	− 10.5	− 30.0	4.5	L thalamus proper
		3.64	− 12.0	− 45.0	− 4.5	L lingual gyrus
3	741	4.62	− 4.5	1.5	− 10.5	L cerebral white matter
		3.88	− 12.0	19.5	− 18.0	L medial orbital gyrus
		3.50	4.5	− 4.5	− 1.5	R thalamus proper
		3.49	4.5	1.5	− 9.0	R ventral diencephalon
		3.43	− 19.5	10.5	− 18.0	L cerebral white matter
		3.41	− 9.0	31.5	− 22.5	L gyrus rectus
		3.33	− 9.0	42.0	− 22.5	L gyrus rectus
		3.24	1.5	4.5	− 1.5	R cerebral white matter
4	556	5.08	− 40.5	22.5	− 12.0	L cerebral white matter
		4.25	− 33.0	27.0	− 19.5	L posterior orbital gyrus
		4.05	− 24.0	30.0	− 15.0	L posterior orbital gyrus
5	544	4.57	− 27.0	− 48.0	− 7.5	L lingual gyrus
		3.96	− 31.5	− 34.5	− 15.0	L parahippocampal gyrus
		3.78	− 39.0	− 18.0	− 22.5	L fusiform gyrus
6	410	5.26	− 33.0	− 90.0	− 4.5	L inferior occipital gyrus
		4.62	− 25.5	− 96.0	1.5	L inferior occipital gyrus
		4.48	− 42.0	− 84.0	− 1.5	L inferior occipital gyrus
		4.01	− 25.5	− 94.5	− 7.5	L cerebral white matter
7	370	5.59	46.5	10.5	− 30.0	R temporal pole
8	258	5.51	− 24.0	− 1.5	− 21.0	L amygdala
9	148	5.26	27.0	− 4.5	− 45.0	R fusiform gyrus
10	116	3.76	25.5	10.5	− 40.5	R temporal pole
11	68	3.86	55.5	3.0	− 13.5	R superior temporal gyrus
12	42	4.00	− 10.5	− 96.0	− 13.5	L lingual gyrus
13	31	3.84	− 19.5	− 69.0	− 9.0	L lingual gyrus
14	20	4.32	10.5	28.5	− 22.5	R gyrus rectus
15	20	4.37	− 31.5	12.0	10.5	L anterior insula

a Reported *t*-values are the peak values after TFCE-based adjustment.

b Labels according to a maximum probability tissue atlas in CAT12 derived from scans originating from the OASIS project with labelled data provided by Neuromorphometrics, Inc. (http://neuromorphometrics.com/), L = left, R = right.

**Fig. 2:**
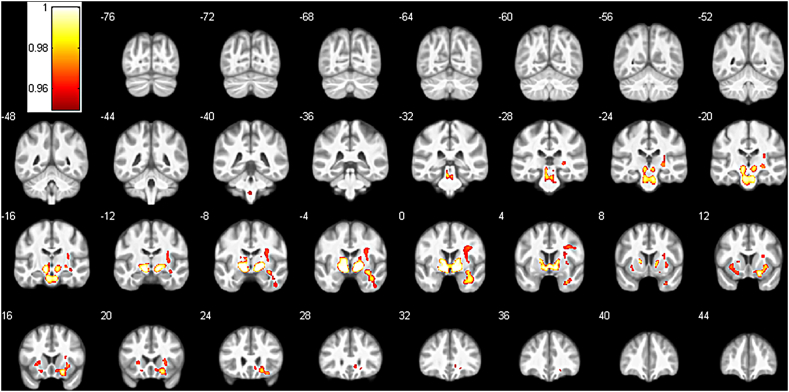
Areas of significantly reduced white matter in the CFS group compared to the control group. Results show the FWE-corrected p-value map (1-p, p < 0.05) overlaid on the average of all normalized images of the entire sample. Numbers indicate y-coordinate of the slice in MNI space.

Significant voxel-wise differences were also seen in a number of white matter regions (see Fig. 2, Table 3). Patients showed reduced WM compared to controls in bilateral areas of the internal and external capsule and anterior midbrain, extending caudally into the bilateral pons, dorsally into the right prefrontal lobe and anteriorly into inferior frontal lobe WM. Additional areas of reduced WM were seen in anterior parts of the right temporal lobe. No areas showed increased WM in the CFS group compared to the control group.

**Table 3:** VBM white matter results.

Cluster index	n Vox	Pseudo-*t*	x	y	z	Labels^a^
1	9640	7.96	− 12	− 1.5	− 7.5	L internal capsule
		7.07	12	− 1.5	− 6	R internal capsule
		6.88	21	13.5	− 15	R inferior fronto-occipital fasciculus
		5.89	9	− 19.5	− 25.5	R corticospinal tract
		5.61	− 6	− 18	− 25.5	L corticospinal tract
		5.18	1.5	3	− 3	Anterior commissure
		4.69	31.5	13.5	− 4.5	R external capsule
		4.58	− 30	12	− 4.5	L external capsule
		4.39	36	3	31.5	R precentral gyrus
		4.24	− 21	13.5	− 13.5	L inferior fronto-occipital fasciculus
		4.19	31.5	24	− 16.5	R lateral fronto-orbital gyrus
		4.07	− 24	18	1.5	L external capsule
		4.06	− 25.5	10.5	− 10.5	L inferior fronto-occipital fasciculus
		3.96	− 4.5	− 30	− 19.5	L superior cerebellar peduncle
		3.95	31.5	− 12	9	R external capsule
		3.77	25.5	− 24	0	R stria terminalis
		3.76	25.5	− 21	− 1.5	R cerebral peduncle
		3.7	30	− 6	22.5	R superior corona radiata
		3.7	25.5	19.5	4.5	R external capsule
		3.61	10.5	15	− 12	R putamen
		3.45	31.5	− 3	10.5	R external capsule
		3.22	45	4.5	30	R precentral gyrus
		3.21	27	10.5	15	R external capsule
		3.14	9	25.5	− 6	R genu of corpus callosum
		2.99	19.5	34.5	− 9	R anterior corona radiata
2	1116	6.09	33	− 3	− 13.5	R inferior fronto-occipital fasciculus
		5.75	36	0	− 25.5	R middle temporal gyrus
		4.22	45	− 6	− 39	R inferior temporal gyrus
3	53	4.73	− 1.5	− 40.5	− 51	Medulla oblongata

a Labels based on the CAT12 provided version of the JHU-MNI-ss atlas (Oishi et al., 2009), L = left, R = right.

### 3.1 Relationship to clinical characteristics

Table 4 shows the results of simple (TIV) and multiple regression (GM, WM, CSF volumes) analyses when predicting clinical characteristics based on volumes. These show negative associations between TIV and almost all questionnaire measures of symptom severity. The multiple regression shows these negative associations for GM and CSF volumes, but not WM volume.

**Table 4:** Relationship to clinical characteristics.

	TIV	GM volume	WM volume	CSF volume
FIS total	− 0.354 **0**.**023**	− 0.587 **0**.**011**	0.349 0.138	− 0.420 **0**.**015**
FIS cognitive	− 0.336 **0**.**032**	− 0.663 **0**.**004**	0.399 *0*.*089*	− 0.371 **0**.**030**
FIS physical	− 0.174 0.276	− 0.304 0.206	0.304 0.226	− 0.395 **0**.**031**
FIS social	− 0.400 **0**.**010**	− 0.598 **0**.**009**	0.301 0.139	− 0.411 **0**.**016**
COMPASS-31	− 0.261 *0*.*099*	− 0.333 0.163	0.230 0.352	− 0.395 **0**.**030**
CFQ	− 0.483 **0**.**001**	− 0.689 **0**.**002**	0.340 0.114	− 0.500 **0**.**002**
Duration of symptoms	− 0.020 0.901	− 0.384 *0*.*096*	0.132 0.579	0.242 0.159
Age at onset	0.266 *0*.*089*	− 0.139 0.545	0.213 0.377	0.301 *0*.*087*

Standardized regression coefficients when predicting clinical measures based on segment volumes in simple regression (TIV) or multiple regression analyses (GM, WM, CSF volumes). In the case of TIV the coefficient is numerically identical to the Pearson correlation between the two variables.

p -value in bold for p < 0.05 and in italics for p < 0.1.

## 4 Discussion

The present study investigated whole-brain and voxel-wise grey matter and white matter volume differences between patients with CFS and healthy controls. Compared to previous studies of volumetric differences (Barnden et al., 2011, Barnden et al., 2016, de Lange et al., 2005, Okada et al., 2004, Puri et al., 2012), with n = 42 the current study investigated one of the largest CFS patient samples to date. Only the recent study by van der Schaaf and colleagues reported findings from a larger sample of CFS patients (van der Schaaf et al., 2016). However, this study focused almost exclusively on GM.

Whole-brain volume differences were seen in white matter with patients showing reduced WM volumes, after accounting for the difference in TIV. We therefore do not replicate the findings of de Lange and colleagues (de Lange et al., 2005) who reported reduced GM volumes in two small patient cohorts, but no difference in WM volume. On the contrary, relative GM volume was larger in patients in the current sample. Even restricting the current analysis to only female participants, as de Lange and colleagues had done, we continue to find a significant reduction in WM, but not GM volume. The trend-level reduction in CSF volume and the significant reduction in TIV, however, disappear. Given that the same group recently also failed to replicate their own findings of GM reduction (de Lange et al., 2005) in a much larger sample, which was otherwise highly comparable (van der Schaaf et al., 2016), the earlier finding of reduced overall GM volume was perhaps a chance finding.

Global WM volume reductions were also reported in a more recent study, which restricted its analysis to supratentorial WM (Zeineh et al., 2015). While it is unclear if the current study would still have found differences in WM volume if the infratentorial region had been excluded from the analysis, the fact that the largest areas of regional WM differences in the voxel-based analysis were supratentorial suggests that this would be the case.

Within the patient group, several subjective measures of symptom severity were related to TIV, with increased symptoms being associated with smaller TIV. This relationship appeared to be driven by the associations with (absolute) GM and CSF volumes. This suggests that CFS patients with smaller TIV are vulnerable to experiencing more severe symptoms. Given the weak positive association at statistical trend level between age at onset and TIV one could speculate that an earlier illness onset may have caused reduced head growth during adolescence and also more severe symptoms. However, age at onset was completely unrelated to any of the symptom measures (all | r | < 0.151, not previously shown). Given that no previous brain volumetric study in CFS has reported either group differences to healthy participants in TIV or associations of TIV with symptom severity, these findings in the present study should be interpreted with caution.

Regional GM volume differences were found in the right insular cortex, right temporal gyrus, bilateral amygdala, left medial temporal lobe and the left lateral occipital lobe and others. However, perhaps surprisingly, the GM volume in all of these regions was increased in the patient group compared to the controls. This stands in contrast to previous findings of GM reductions in prefrontal areas (Okada et al., 2004) and parahippocampal and occipital cortex (Puri et al., 2012). However, cortical thickness has been shown to be increased in CFS patients in several right hemisphere regions in one recent study, particularly for younger individuals (Zeineh et al., 2015). Other studies failed to show regional GM differences, although several of these studies used overall GM segment volume as a covariate in their group comparison, which may have reduced the power to detect regional differences, as systematic differences between groups in local GM volume would be partially reflected in overall GM volume as well and may thereby mask regional differences (Henley et al., 2010).

Of the regional GM differences that were found in the current study, the two large areas involving the amygdala with the right one extending into the insular cortex appear to be of most direct relevance to CFS symptomatology. The insula is involved in a variety of functions, including interoceptive (Strigo and Craig, 2016) but also cognitive and affective functions and is directly connected to subcortical targets such as the amygdala (Shura et al., 2014). The amygdala has also been linked to a variety of different functions, including emotional processing, fear conditioning and memory processes. Enlarged amygdala volume has been shown in individuals with joint hypermobility (Eccles et al., 2012), an abnormality of connective tissue which has been linked to CFS and similar conditions (Eccles et al., 2015, Nijs et al., 2006). However, as joint hypermobility was not assessed in the current study the degree to which it may have played a role the present findings remains unknown.

Previous models of CFS have hypothesised a role for the amygdala in the pathophysiology of the condition (Gupta, 2002, Wyller et al., 2009). Its general role is perhaps best described as a salience and valence detector (Benarroch, 2015). It evaluates whether or not incoming sensations are of potential consequence for the equilibrium of the organism (salience) and whether any such disturbances are negative or positive (valence). The observed GM changes in the amygdala, together with the changes seen in the insula, could therefore suggest altered processing and evaluation, particularly of interoceptive signals in patients with CFS with consequences for both autonomic responses and cognitive/affective processing. Two recent studies showed reduced resting-state functional connectivity in CFS patients in the insula (Boissoneault et al., 2016, Wortinger et al., 2016). Previous findings of GM volume reductions in dorsolateral prefrontal cortex (DLPFC) may further support this notion. The DLPFC and the amygdala are part of a larger network of brain regions that is involved in the processing of emotional information and stress (Comte et al., 2016, Sinha et al., 2016). The observed changes in structure and function in these areas across different studies therefore support the hypothesis that CFS is associated with alterations to this cortico-limbic system.

The identified GM differences in the other areas are more difficult to interpret given their location and extent. In general, areas around the superior temporal sulcus are associated with speech and auditory functions (Alho et al., 2014, Zaehle et al., 2008), though they tend to be more lateralized to the left side, not the right. However, the GM increases are adjacent to areas of white matter that has been shown to be abnormal in CFS in our own and in a previous study (Zeineh et al., 2015). Similarly, it is unclear how the cluster we identified in the lateral occipital lobe could be relevant for CFS, as it appears to incorporate cortical areas that are primarily concerned with various aspects of basic visual processing (Kolster et al., 2010).

The locations of regional white-matter abnormalities that were identified in the current study appear to overlap considerably with regional WM changes seen in other studies. The current study identified reduced WM in an area of the anterior temporal lobe, which appears to belong to the uncinate fasciculus (UF). A recent DTI study (Zeineh et al., 2015) showed that CSF patients had increased fractional anisotropy (FA) in the anterior part of the right inferior longitudinal fasciculus (ILF), which they argued could be due to a reduction in crossing fibres. Importantly, ILF and UF meet in the anterior portion of the temporal lobe. The observed WM volume reductions in the UF from the current study may therefore indirectly support the hypothesis of reduced crossing fibres as an explanation for the increase in FA in the ILF observed in this earlier study (Zeineh et al., 2015).

Other areas of reduced WM in the current study were those seen in the midbrain and brainstem. The midbrain cluster shows some resemblance to areas identified by others, who reported WM volume changes in a large parts of the midbrain (Barnden et al., 2011). However, instead of a significant group difference the previous study showed a significant negative correlation between WM volume and fatigue duration in patients. Given this strong correlation, it is surprising that no group difference was found, if one considers controls as individuals with zero (chronic) fatigue duration. The two areas also don't seem to fully overlap, with the one from the present study being located slightly more anteriorly and more dorsally. Nonetheless, similar WM tracts appear to be affected in both studies.

It is possible that the area of reduced WM in the ventral pons consists of the same WM tracts that already showed reductions further ventrally. Barnden and colleagues (Barnden et al., 2011) also identified a large cluster involving the brainstem and cerebellum that showed a differential association between T1-weighted signal intensity and seated pulse pressure in the CFS compared to the healthy control group. They hypothesised altered cerebral autoregulation via astrocyte dysfunction as a potential cause for their pattern of results. If impaired cerebral autoregulation is indeed causing the effects observed by Barnden and colleagues, this could ultimately result in tissue damage and volume reductions. The differences in methodology and the larger sample size of the current study may account for the fact that the previous study did not find a group difference in volume, whereas the current study did.

Several limitations of the current study need to be acknowledged. First, although one of the largest studies to date that investigates brain morphometric differences in CFS, the sample size of the present study can still only be considered moderate. This is particularly important given the heterogeneity of symptom presentation and illness progression in CFS. Larger samples, perhaps from collaborative studies, will be necessary to further elucidate the role of altered brain structure and function in CFS. Ideally such studies should consider longitudinal designs, as the currently available studies, which are almost exclusively cross-sectional, will be unable to distinguish potential causes from consequences of CFS. Second, in the current study no diffusion weighted images were available to further elucidate the apparent volumetric changes in WM. Thus, it is unclear if the volumetric differences seen in the current study correspond to changes in WM microstructure. Multimodal imaging approaches such as those presented by others (Barnden et al., 2011, Barnden et al., 2016, Zeineh et al., 2015) should be performed whenever possible to more comprehensively characterise any changes to brain structure in CFS. Lastly, it should be noted that, at the time of writing, the CAT12 toolbox (Gaser and Dahnke, 2016), which was used for segmentation and normalization, had not yet been formally evaluated.

## 5 Conclusion

The current study showed alterations in both grey matter and white matter volume in patients with Chronic Fatigue Syndrome. Some of those findings, particularly those in GM, are novel and may indicate altered processing of interoceptive signals in CFS patients. Future studies should incorporate measures of interoceptive processing to more directly interrogate this potential link to CFS. The differences in WM volume were seen in areas that have previously shown abnormalities in CFS albeit partially of a different kind. The current findings suggest abnormalities in a network of insular-limbic regions, as well as changes to WM tracts of the midbrain, brainstem and temporal cortex.

## Conflicts of interest and source of funding

The authors declare no conflicts of interest. This research was funded by the Medical Research Council (MR/J002712/1). AF is supported by Research Capability Funding from the Newcastle upon Tyne Hospitals NHS Foundation Trust and the Northumberland, Tyne and Wear NHS Foundation Trust.

## Appendix A Supplementary data

Supplementary material 1: GM results, CFS>HC, (1-p)-map (FWE). Image 1

Supplementary material 2: WM results, CFS<HC, (1-p)-map (FWE). Image 2

Supplementary material 3: mean normalized T1 image. Image 3
